# Preliminary Proteomic Analysis of A549 Cells Infected with Avian Influenza Virus H7N9 and Influenza A Virus H1N1

**DOI:** 10.1371/journal.pone.0156017

**Published:** 2016-05-25

**Authors:** Xiaoman Ding, Jiahai Lu, Ruoxi Yu, Xin Wang, Ting Wang, Fangyuan Dong, Bo Peng, Weihua Wu, Hui Liu, Yijie Geng, Renli Zhang, Hanwu Ma, Jinquan Cheng, Muhua Yu, Shisong Fang

**Affiliations:** 1 School of Public Health, Sun Yat-sen University, Guangzhou, China; 2 Southern Medical University, Guangzhou, China; 3 Major Infectious Disease Control Key Laboratory, Key Reference Laboratory of Pathogen and Biosafety, Shenzhen Center for Disease Control and Prevention, Shenzhen, China; 4 Nanshan Center for Disease Control and Prevention, Shenzhen, China; Centers for Disease Control and Prevention, UNITED STATES

## Abstract

A newly emerged H7N9 influenza virus poses high risk to human beings. However, the pathogenic mechanism of the virus remains unclear. The temporal response of primary human alveolar adenocarcinoma epithelial cells (A549) infected with H7N9 influenza virus and H1N1 influenza A virus (H1N1, pdm09) were evaluated using the proteomics approaches (2D-DIGE combined with MALDI-TOF-MS/MS) at 24, 48 and 72 hours post of the infection (hpi). There were 11, 12 and 33 proteins with significant different expressions (*P<*0.05) at 24, 48 and 72hpi, especially F-actin-capping protein subunit alpha-1 (CAPZA1), Ornithine aminotransferase (OAT), Poly(rC)-binding protein 1 (PCBP1), Eukaryotic translation initiation factor 5A-1 (EIF5A) and Platelet-activating factor acetylhydrolaseⅠb subunit beta (PAFAH1B2) were validated by western-blot analysis. The functional analysis revealed that the differential proteins in A549 cells involved in regulating cytopathic effect. Among them, the down-regulation of CAPZA1, OAT, PCBP1, EIF5A are related to the death of cells infected by H7N9 influenza virus. This is the first time show that the down-regulation of PAFAH1B2 is related to the later clinical symptoms of patients infected by H7N9 influenza virus. These findings may improve our understanding of pathogenic mechanism of H7N9 influenza virus in proteomics.

## Introduction

Since March 2013, A novel H7N9 influenza virus first crossed the species barrier and infected humans in East China, causing disease with unusually high morbidity and mortality. It is a reassortant virus which consists of the gene fragments from earlier H7N9, H7N3 and H9N2 viruses [1–3]. Human infection can be caused by the direct contact with the poultry infected with H7N9, since most of infected persons have ever exposed to the live poultry [4]. The patients had common symptoms during the early stage and later suffered from severe clinical outcomes including severe pneumonia, acute respiratory distress syndrome (ARDS), multi-organ dysfunction and encephalopathy [1,5–7].

In recent years, many new discoveries have been made concerning H7N9 infection. Sakai et al [8] demonstrated that recently emerged H7N9 as well as seasonal IAVs mainly used the specific protease TMPRSS2 for HA cleavage in vivo. Viruses H5N1, H7N7, and H7N9 were pathogenic in mice, Morrison et al [9] found that this pathogenicity was correlated with increased transcription of cytokine response genes and decreased transcription of lipid metabolism and coagulation signaling genes. To the best of our knowledge, few proteome analyses have been reported comparing the differences in the cellular host response between H7N9 and H1N1pdm09 viruses at the proteomic level, especially in the whole process of infection. It is clear that the pandemic of the H1N1pdm09 is relatively mild. Unlike the H1N1pdm09, the H7N9 influenza virus has never been extensively circulated among the humans, so the lacking of preexisting immunity presents the human population at high risk [10,11]. Noteworthily, our previous studies [12] and available data [13–15] suggested that the H1N1 infection induced less of a response. There was no obvious protein profile changes of cells including A549 cells infected with H1N1 virus. In addition, A549 cells are widely used for the investigation of influenza A virus replication in vitro or in other proteome studies. The avian influenza virus generally prefer α-2,3 sialic acid receptors. The H7N9 virus can invade the lower respiratory tract of human beings because the virus retain strong affinity for the α-2,3 sialic acid receptors present in the lower respiratory tract of human [16,17]. Notably, the virus titre in lung tissues was about tenfold higher than that in tracheal tissues [11]. Therefore, We selected H1N1 pdm09-infected A549 cells as the control group in this study. The fluorescent two dimensional difference gel electrophoresis (2D-DIGE) and MALDI-TOF–MS/MS were applied to investigate the difference in host proteome after infection with the two influenza virus strains and explored the underlying pathogenic mechanism of H7N9 infection in mammals.

## Materials and Methods

### Ethics statement

All procedures performed in studies were in accordance with the ethical standards of the institutional and/or national research committee and with the Helsinki declaration and were approved by the Medical Ethics Committee of Shenzhen Center for Disease Control and Prevention (SZCDC), Shenzhen, P. R. China.

### Reagents

A549 cell line, H7N9 and H1N1pdm09 influenza virus were obtained from Shenzhen Center for Disease Control and Prevention (SZCDC). All the reagents for DIGE were purchased from GE Healthcare (Pittsburgh, PA, USA). DL-Dithiothreitol (DTT), Iodoacetamide (IAA), CHAPS were purchased from Sigma–Aldrich (Louis, MO, USA). The antibodies against CAPZA1, OAT, PCBP1, EIF5A, PAFAH1B2 were purchased from Abcam (Cambridge, MA, USA). The antibody against β-action was purchased from Santa Cruz (Santa Cruz, CA, USA). The secondary antibodies of goat anti-mouse and goat anti-rabbit were purchased from Thermo Fisher (Rockford, IL, USA). The restriction enzyme (BamH I and Not I) were purchased from NEB(USA).

Lipofectamine2000 and pcDNA3.1/Neo(+) were purchased from Invitrogen (Carlsbad, CA, USA).

### Cell culture and virus infection

A549 cells were cultured in Dulbecco’s modified Eagle’s medium (DMEM) supplemented with 10% fetal bovine serum (FBS) and incubated at 37°C under 5% CO_2_. To ensure most of the cells were able to grow adhering to the wall at 72hpi, a multiplicity of infection (MOI) of 0.001 was used [18]. Cells were infected with H7N9 or H1N1pdm09 influenza virus in independent biological triplicates and the cell culture supernatants including 0.25μg/mL of trypsin. All experiments with H7N9 influenza virus were performed in enhanced biosafety level 3 (BSL3) containment laboratories at SZCDC.

### Protein extraction, quantification and labeling

At set time points post-infection (24, 48, and 72h), medium was aspirated, cells were washed three times by PBS and then lysed on ice with lysis buffer (7M urea, 2M thiourea, 30mM Tris-Hcl PH 8.8, 4% w/v CHAPS) for 30min. All the cells were then scraped into a 1.5mL eppendorf centrifuge tube and sonicated for 30s, centrifuged at 14,000×g for 60 min at 4°C. Purification and quantification of the proteins were performed strictly according to the instruction of 2-D Clean-Up Kit and 2-D Quant Kit (GE healthcare).

The biological replicates at set time points were labeled and reversely labeled by 200pmol Cy3 and Cy5 fluorescence dyes from CyDye DIGE Fluor Minimal Labeling Kit (GE healthcare), respectively. A total of 25ug of all individual samples were pooled as internal standard and labeled by 200pmol Cy2 dye. The labeling reaction was carried out on ice in darkness for 30 min and then quenched with 1uL of 10 mM lysine for 10 min. For each gel, Cy2-, Cy3-, and Cy5-labeled proteins (25ug each) were combined and an equal volume of 2×sample buffer (7M urea, 2M thiourea, 4% w/v CHAPS, 2% w/v DTT, 2% v/v IPG buffer pH 3–11) was added, then brought the total volume of the sample to 450uL with rehydration buffer (8M urea, 2% w/v CHAPS, 0.5% v/v IPG buffer pH 3–11, 0.5% w/v DTT, 0.002% w/v bromophenol blue.

### 2-D electrophoresis

Isoelectric focusing (IEF) was carried out using the Ettan IPGphor focusing apparatus (GE Healthcare) and the extracted proteins were loaded onto 24cm, 3-11NL immobilized pH gradient (IPG) strips (GE healthcare). The IEF procedure was as follows: 50V 18h, 300V 2h, 500V 2h, 1000V 2h, gradient 8000V 8h, 8000V 8h. After IEF, the IPG strips were incubated in equilibration buffer (6M urea, 75mM Tris–Hcl PH 8.8, 29.3% v/v glycerol, 2% w/v SDS, 0.002% w/v bromophenol blue) supplemented with 1% w/v DTT for 15min. This step was repeated using the same buffer with 2.5% w/v IAA in place of 1% DTT. The strips were then put on the top of 12.5% SDS-PAGE gels with 1W per gel for 1h and 11W per gel for about 6h at 15°C.

### Gel image acquisition and analysis

After 2-D electrophoresis, the gel images for analysis were obtained by using the Typhoon TRIO Imager (GE Healthcare) and were processed in DeCyder 6.5 differential analysis software (GE Healthcare). To assess the biological variation, three individual infection experiments were carried out at three time points and three gel replicates were used for inter-gel matching performed with the Biological Variation Analysis (BVA) software module. These proteins were found to be different expressed between H7N9- and H1N1pdm09-infected A549 cells using a Student’s t-test. Protein spots with significant differences in abundance (H7N9/H1N1 over 1.5-folds, *P*<0.05) were selected for mass spectrometry.

### Protein identification by in-gel-digestion and MALDI-TOF–MS/MS

Interested protein spots from the Coomassie-stained gels were manually excised. The gel samples were placed in a 1.5mL eppendorf centrifuge tube and washed twice with 500uL ddH_2_O. The gel pieces were destained and dehydrated, and then digested using sequencing-grade trypsin (Promega, Madison, WI, USA) overnight at 37°C. The resulting peptides were then analyzed by an Ultrafle Xtreme MALDI-TOF/TOF Mass Spectrometer (Bruker Daltonics Inc, Billerica, MA, USA). The peptide mass fingerprint (PMF) combined MS/MS data were submitted to MASCOT version 2.2 (Matrix Science, Boston, MA, USA) for identification according to the SwissProt database. The parameters were set as follows: Homo sapiens, trypsin cleavage, missed cleavage, carbamidomethylation as a fixed modification, methionine oxidation as a variable modification, peptide mass tolerance set at 100ppm, and fragment tolerance set at 0.5Da. Proteins matching more than two peptides and protein scores > 30 were considered statistically significant (*P*<0.05).

### Bioinformatics analysis

Protein ontology classification was performed with PANTHER classification system (http://www.pantherdb.org/) by importing into the accession of the proteins. The differentially expressed proteins at 24, 48, 72hpi were grouped according to their protein classes.

The String software (http://string-db.org/) was utilized to perform a protein-protein interaction network for the differentially expressed proteins at 24, 48, 72hpi by searching the String database and the protein-protein interaction network database.

### Western blot analysis

The proteins from A549 cells infected with H7N9 and H1N1pdm09 influenza virus at 24, 48, 72hpi as well as time-matched mock-infected cells were extracted and quantified using a BCA assay (Thermo Fisher Scientific). Total proteins were separated by one-dimensional SDS-PAGE and transferred to PVDF membranes. Membranes were blocked with 5% (w/v) nonfat dry milk in TBST and subsequently incubated overnight at 4°C with the corresponding primary antibodies anti-β-action (1:1000), anti-CAPZA1 (1:10000), anti-OAT (1:1000), anti-PCBP1 (1:1000), anti-EIF5A (1:5000), anti-PAFAH1B2 (1:1000). After three washes with TBST, membranes were incubated for 90 min at room temperature with horseradish peroxidase labeled secondary antibodies (1:5000). Membranes were then washed thoroughly with TBST, scanned using Image Quant LAS4000 (GE healthcare), and analyzed quantitatively.

### The expression of PAFAH1B2 in H7N9 infection

The Genbank accession number of PAFAH1B2 is CR456736 and the cDNA (690bp) was synthesized artificially. The PCR primers (sense:5’-GCGGGATCCAT GAGCCAAGGAGACTCAAACCCA-3’;antisense:5’-GCGGCGGCCGCTTAAGCA ATGGTGGTTTGTTTCTCC-3’) based on this fragment were synthesized. We created the PAFAH1B2 cDNA by PCR amplification with primers containing a 5’BamHI restriction site and a 3’NotI restriction site for subcloning into pcDNA3.1/Neo(+) via BamH I and Not I, which created the final vector, pcDNA3.1/Neo-PAFAH1B2. The sequences of all clones were verified by sequence analysis[19–20].

Lipofectamine2000 was employed for the transient transfections of the recombinant eukaryotic expressing plasmids pcDNA3.1/Neo-PAFAH1B2 in A549 cells. At 24 hours post of transfection, the A549 cells with recombinant plasmid were infected by H7N9 influenza virus and observed the levels of PAFAH1B2 at 24,48 and 72hpi by western blot analysis. Besides, the levels of PAFAH1B2 in primary A549 cells and cells with recombinant plasmid were also analyzed by western blot.

## Results

### 2-D DIGE screening and identification of differentially expressed proteins

**Fig 1:**
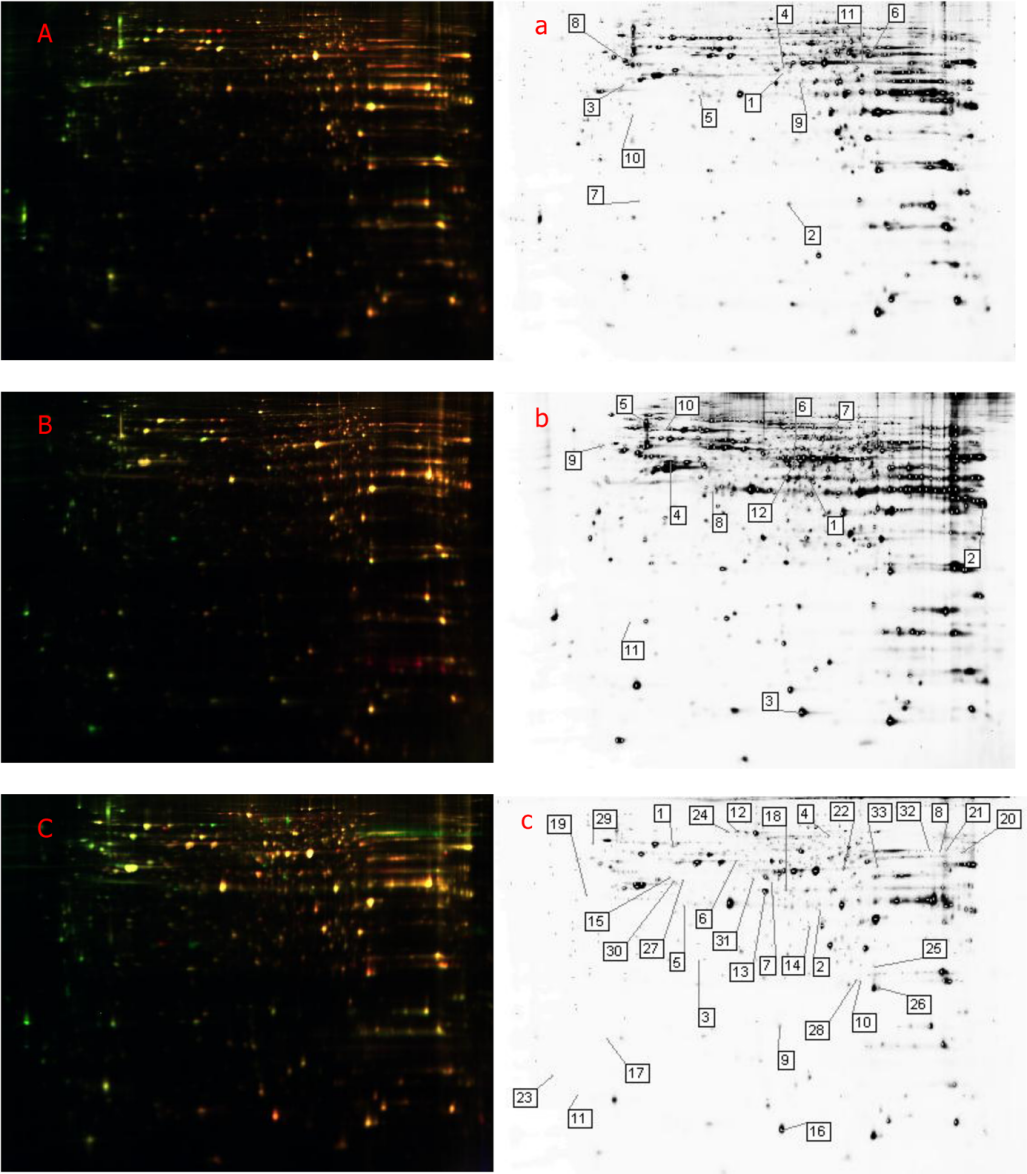
2D- DIGE images and differentially expressed proteins of H7N9- and H1N1pdm09-infected A549 cells. The 2D- DIGE images of A549 cells infected with H7N9 and H1N1pdm09 influenza virus at 24 (A), 48 (B) and 72 (C) hpi. 2D- DIGE analysis of the differentially expressed spots between H7N9- and H1N1pdm09-infected A549 cells at 24 (a), 48 (b) and 72(c) hpi.

DIGE analysis (Fig 1) the differentially expressed spots (Students t-test *P*<0.05). The significantly up- or down-regulated proteins by at least 1.5-folds were subject to the further analysis by MALDI-TOF–MS/MS identification unambiguously. A total of 11,12,33 protein spots were found to be differentially expressed in A549 cells infected with H7N9 and H1N1pdm09 influenza virus at 24, 48, 72hpi, respectively (Tables 1, 2 and 3), especially F-actin-capping protein subunit alpha-1 (CAPZA1), Ornithine aminotransferase (OAT), Poly(rC)-binding protein 1 (PCBP1), Eukaryotic translation initiation factor 5A-1 (EIF5A) and Platelet-activating factor acetylhydrolaseⅠb subunit beta (PAFAH1B2). The data showed that the up- or downregulated of these five proteins were associated with the time of infection by the two strains.

**Table 1:** Identification of differentially expressed proteins in A549 cells infected with H7N9 and H1N1pdm09 influenza virus at 24hpi.

Spots No.	Names	Accession	Score	MW(kDa)/PI	AV.ratio (H7N9/H1N1)	P value(T-test)
*1	Ornithine aminotransferase	OAT_HUMAN	185	48846/6.6	-2.04	0.00054
2	Cofilin-1	COF1_HUMAN	273	18719/9.1	1.72	0.00055
3	Serine-threonine kinase receptor-associated protein	STRAP_HUMAN	248	38756/4.8	-2.14	0.0021
4	Annexin A7	ANXA7_HUMAN	118	52991/5.4	-1.56	0.0038
*5	F-actin-capping protein subunit alpha-1	CAZA1_HUMAN	98	33073/5.4	-2.3	0.0042
6	Retinal dehydrogenase 1	AL1A1_HUMAN	165	55454/6.3	1.64	0.0081
7	Eukaryotic translation initiation factor 1A	IF1AX_HUMAN	244	16564/4.9	-2.13	0.011
8	Tubulin beta-2A chain	TBB2A_HUMAN	346	50274/4.6	-1.6	0.018
*9	Poly(rC)-binding protein 1	PCBP1_HUMAN	56	37987/6.8	-2.13	0.021
10	EF-hand domain-containing protein D2	EFHD2_HUMAN	381	26795/5.0	-1.75	0.022
11	Alpha-enolase	ENOA_HUMAN	145	47481/7.7	1.63	0.041

* Indicating that the differential proteins were validated by western blot analysis.

**Table 2:** Identification of differentially expressed proteins in A549 cells infected with H7N9 and H1N1pdm09 influenza virus at 48hpi.

Spots No.	Names	Accession	Score	MW(kDa)/PI	AV.ratio (H7N9/H1N1)	P value(T-test)
*1	Poly(rC)-binding protein 1	PCBP1_HUMAN	237	37987/6.8	-1.82	0.00026
2	Heterogeneous nuclear ribonucleoprotein A1	ROA1_HUMAN	378	38837/9.6	-1.57	0.0018
3	U6 snRNA-associated Sm-like protein LSm2	LSM2_HUMAN	97	10942/6.1	1.73	0.0018
4	Actin, cytoplasmic 1	ACTB_HUMAN	349	42052/5.2	-1.72	0.0041
5	Vimentin	VIME_HUMAN	362	53676/4.9	-2.6	0.0047
*6	Ornithine aminotransferase	OAT_HUMAN	244	48846/6.6	-2.09	0.0048
7	Pyruvate kinase isozymes M1/M2	KPYM_HUMAN	171	58470/9.0	-2.31	0.0049
*8	F-actin-capping protein subunit alpha-1	CAZA1_HUMAN	33	33073/5.4	-3.26	0.0082
9	UV excision repair protein RAD23 homolog B	RD23B_HUMAN	65	43202/4.6	-4.33	0.0098
10	Heat shock cognate 71 kDa protein	HSP7C_HUMAN	183	71082/5.2	-2	0.01
*11	Eukaryotic translation initiation factor 5A-1	IF5A1_HUMAN	45	17049/4.9	-4.5	0.011
12	Pyruvate dehydrogenase E1 component subunit alpha	ODPA_HUMAN	83	43952/9.3	-2.64	0.012

* Indicating that the differential proteins were validated by western blot analysis.

**Table 3:** Identification of differentially expressed proteins in A549 cells infected with H7N9 and H1N1pdm09 influenza virus at 72hpi.

Spots No.	Names	Accession	Score	MW(kDa)/PI	AV.ratio (H7N9/H1N1)	P value(T-test)
1	Heat shock 70 kDa protein 1A/1B	HSP71_HUMAN	277	70294/5.4	2.45	2.00E-05
2	Heterogeneous nuclear ribonucleoprotein D-like	HNRDL_HUMAN	56	46580/10	-2.57	3.60E-05
*3	Platelet-activating factor acetylhydrolase Ⅰb subunit beta	PA1B2_HUMAN	138	25724/5.5	-15.52	9.80E-05
4	Prelamin-A/C	LMNA_HUMAN	150	74380/6.6	2.26	0.00014
*5	F-actin-capping protein subunit alpha-1	CAZA1_HUMAN	192	33073/5.4	-4.27	0.00027
6	Heterogeneous nuclear ribonucleoprotein H	HNRH1_HUMAN	392	49484/5.9	1.66	0.00028
*7	Ornithine aminotransferase	OAT_HUMAN	38	48846/6.6	-2.95	0.00029
8	Peptidyl-prolyl cis-trans isomerase B	PPIB_HUMAN	50	23785/9.9	-8.39	0.00065
9	Cofilin-1	COF1_HUMAN	213	18719/9.1	-1.6	0.0008
10	Transgelin-2	TAGL2_HUMAN	46	22548/9.3	1.97	0.0018
11	Transcription elongation factor B polypeptide 1	ELOC_HUMAN	71	12636/4.6	-3.53	0.0027
12	Ezrin	EZRI_HUMAN	107	69484/5.9	1.59	0.0028
13	Macrophage-capping protein	CAPG_HUMAN	155	38760/5.8	1.53	0.0031
14	Proteasome subunit alpha type-1	PSA1_HUMAN	230	29822/6.2	1.52	0.0044
15	Thioredoxin domain-containing protein 5	TXND5_HUMAN	38	48283/5.6	-2.25	0.0058
16	U6 snRNA-associated Sm-like protein LSm2	LSM2_HUMAN	119	10942/6.1	1.93	0.0058
*17	Eukaryotic translation initiation factor 5A-1	IF5A1_HUMAN	112	17049/4.9	-5.96	0.0072
*18	Poly(rC)-binding protein 1	PCBP1_HUMAN	72	37987/6.8	1.72	0.0078
19	Nucleophosmin	NPM_HUMAN	208	32726/4.5	-3.33	0.0083
20	ATP synthase subunit alpha	ATPA_HUMAN	122	59828/9.6	-2.61	0.0087
21	Aconitate hydratase, mitochondrial	ACON_HUMAN	333	86113/7.9	-3.57	0.0088
22	Alpha-enolase	ENOA_HUMAN	160	47481/7.7	1.97	0.009
23	Myosin light polypeptide 6	MYL6_HUMAN	120	17090/4.4	-3.29	0.0092
24	Gelsolin	GELS_HUMAN	164	86043/5.9	1.6	0.013
25	Flavin reductase (NADPH)	BLVRB_HUMAN	41	22219/7.9	1.5	0.014
26	Phosphatidylethanolamine-binding protein 1	PEBP1_HUMAN	209	21158/7.8	1.58	0.016
27	Actin, aortic smooth muscle	ACTA_HUMAN	51	42381/5.1	2.25	0.019
28	Superoxide dismutase [Mn]	SODM_HUMAN	119	24878/9.1	4.23	0.019
29	Microfibrillar-associated protein 1	MFAP1_HUMAN	65	51927/4.8	-2.19	0.021
30	Protein NDRG1	NDRG1_HUMAN	172	43264/5.4	1.69	0.021
31	TAR DNA-binding protein 43	TADBP_HUMAN	56	45053/5.8	1.61	0.023
32	Retinal dehydrogenase 1	AL1A1_HUMAN	95	55454/6.3	-8.89	0.029
33	Vasodilator-stimulated phosphoprotein	VASP_HUMAN	114	39976/9.7	1.68	0.036

* Indicating that the differential proteins were validated by western blot analysis.

### Protein classes and protein interaction network diagram

**Fig 2:**
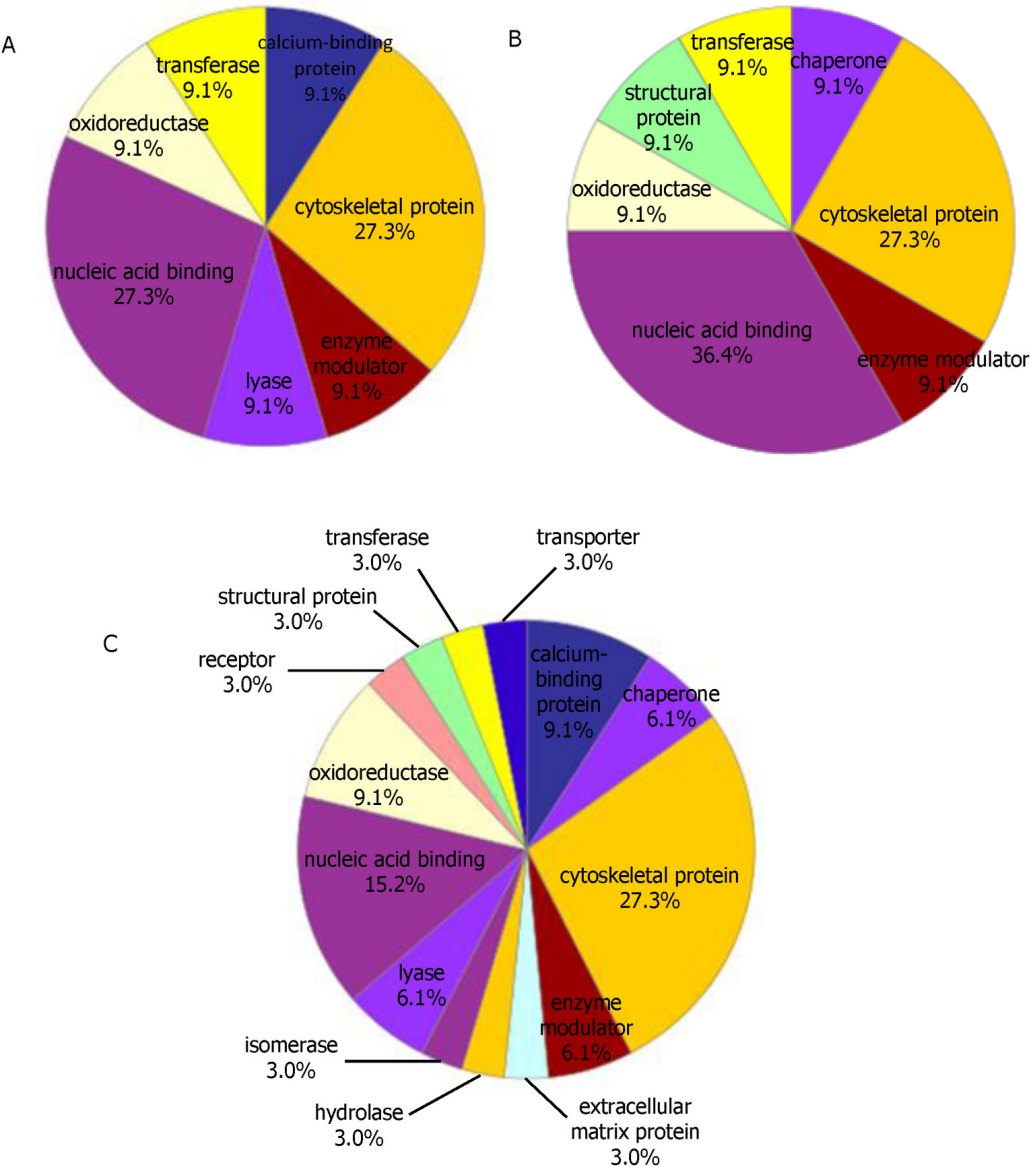
**Functional classifications of the identified proteins according to their protein classes of 24h(A), 48h (B) and 72h (C) by PANTHER**.

The differentially expressed proteins in A549 cells infected with the two strains at 24, 48, 72hpi were imported into the PANTHER database. The PANTHER classification system revealed that the proteins could be classified into groups according to their protein classes. Most of the differential proteins at 24hpi belonged to nucleic acid binding (27.3%), cytoskeletal protein (27.3%), transferase (9.1%), lyase (9.1%), calcium-binding protein (9.1%), enzyme modulator (9.1%) and oxidoreductase (9.1%). More classes of the differential proteins (receptor, structural protein, transporter, chaperone, isomerase, hydrolase, extracellular matrix protein) were expressed in A549 cells in the later phase of infection (Fig 2).

**Fig 3:**
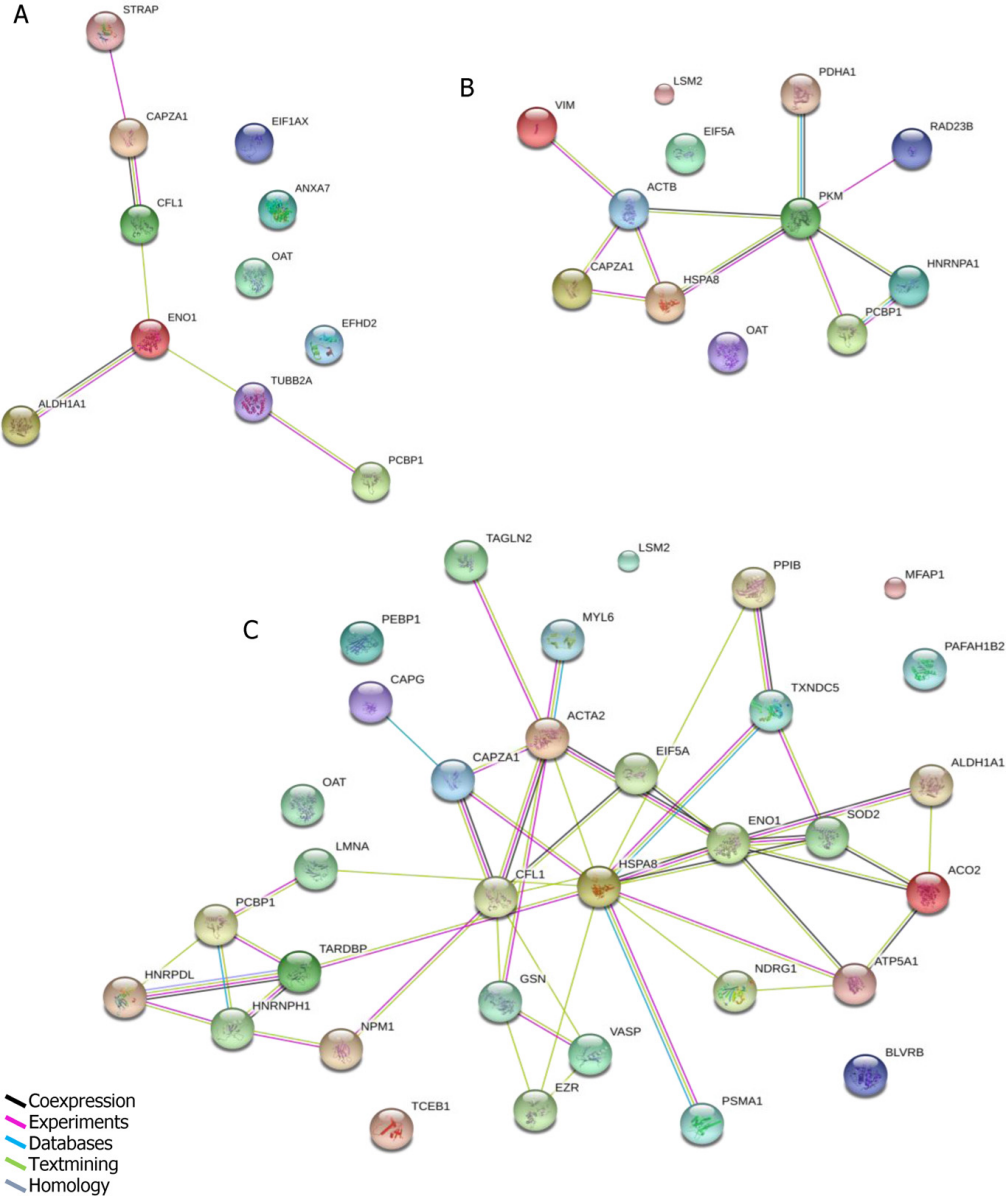
**The protein interaction network diagrams of 24h(A), 48h(B)and 72h(C)were analyzed by String software.** Colored lines denote interactions.

The protein interaction network diagram was constructed for the differentially expressed proteins in A549 cells infected with the two virus strains at 24, 48, 72hpi. As shown in (Fig 3),the protein interaction network diagram became more and more complicated during the time of infection. The proteins CAPZA1, PCBP1, and EIF5A were located in the central area of the network. However, OAT and PAFAH1B2 were not included in the network, they also played a crucial role in the process of H7N9 infection.

### Validation of the differentially expressed proteins by western-blot analysis

**Fig 4:**
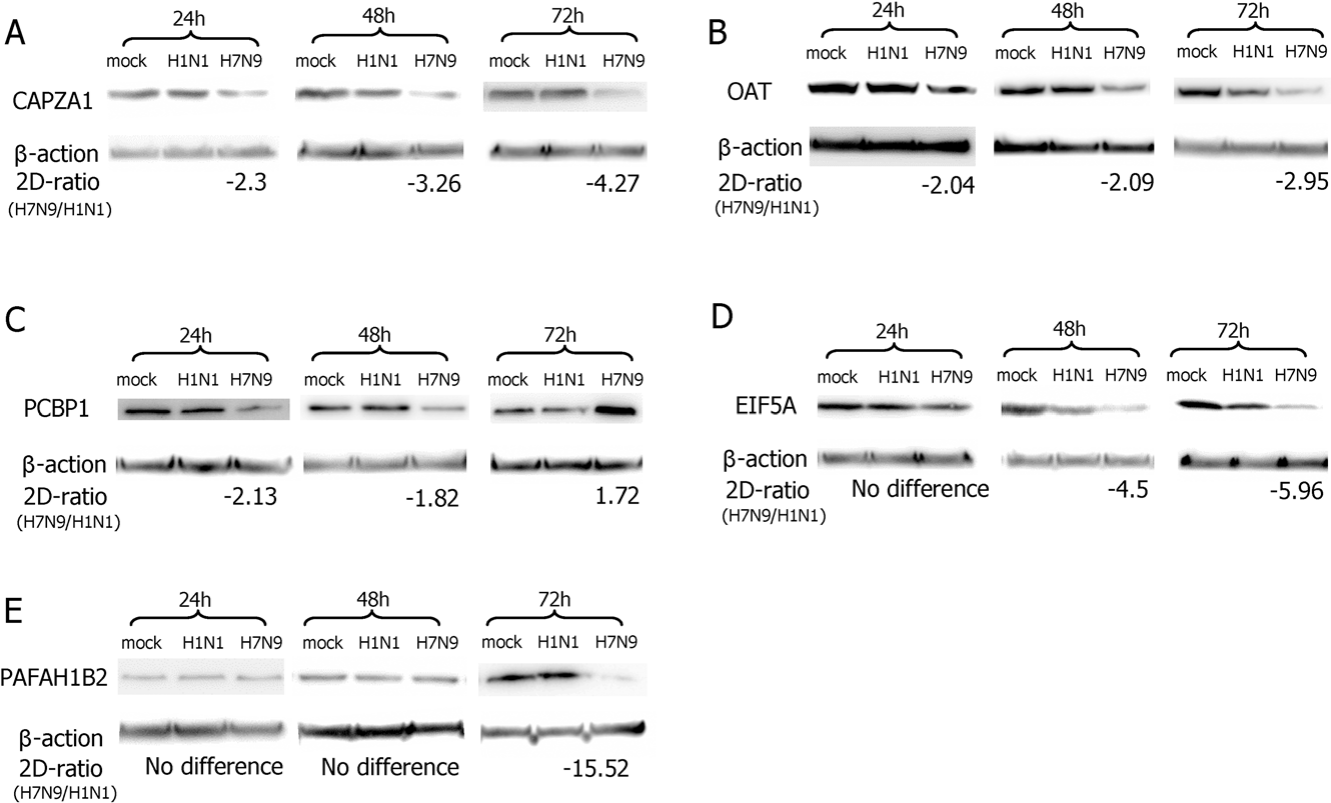
Results of western blot analysis. The differentially expressed proteins of CAPZA1 (A), OAT (B), PCBP1 (C), EIF5A (D), PAFAH1B2 (E) in H7N9- and H1N1pdm09-infected A549 cells at 24, 48 and 72hpi as well as time-matched mock-infected cells were confirmed by western blot analysis.

In order to validate the data obtained from 2-D DIGE and MALDI-TOF–MS/MS, we performed western blots. In 3 independent western blot analyses (*P*<0.05), as shown in (Fig 4), compared with the H1N1pdm09 infection, the down- or up-regulated proteins of CAPZA1 (33KDa), OAT (49KDa), PCBP1 (37KDa), EIF5A (18KDa), PAFAH1B2 (26KDa) in H7N9 infection were consistent with the 2-D DIGE results. Furthermore, the expression of these five proteins were not affected in the cells infected by H1N1pdm09 virus compared with the time-matched mock infection cells.

### Validate the expression of PAFAH1B2 in H7N9 infection by western-blot analysis

**Fig 5:**
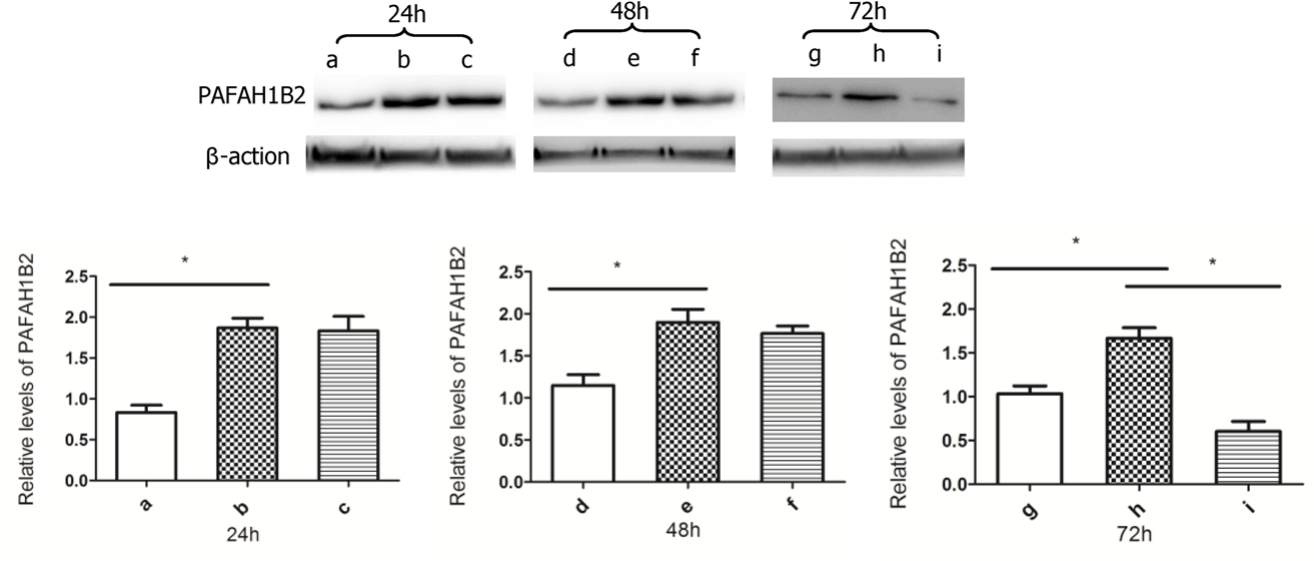
The expression of PAFAH1B2 in H7N9 infection. The *a*, *d* and *g* were the relative levels of PAFAH1B2 in primary A549 cells. The *b*, *e* and *h* were the relative levels of PAFAH1B2 in A549 cells with recombinant plasmid. The *c*, *f* and *i* were the relative levels of PAFAH1B2 in A549 cells with recombinant plasmid infected by H7N9 virus at 24,48 and 72hpi. The date are shown the relative levels of PAFAH1B2 with SD (n = 3,*p<0.05).

The A549 cells with over-expressing PAFAH1B2 were constructed successfully, as shown in (Fig 5A, 5B, 5D, 5E, 5G and 5H).The levels of PAFAH1B2 in A549 cells with recombinant plasmid were about twice than the primary A549 cells. We also observed that the levels of PAFAH1B2 in H7N9-infected A549 cells with recombinant plasmid were not down-regulated obviously until 72hpi compared with the non-infected A549 cells with recombinant plasmid (Fig 5B, 5C, 5E, 5F, 5H and 5I). The results further prove that the H7N9 virus seriously affect the synthesis of PAFAH1B2 in A549 cells in the later phase of infection.

## Discussion

Our proteomic analysis revealed that the intracellular proteins were differentially translated in H7N9- and H1N1pdm09-infected A549 cells at 24, 48, 72hpi.The prominent response was marked by the change in abundance of CAPZA1, OAT, PCBP1, EIF5A and PAFAH1B2.

F-actin capping protein (CapZ) is an αβ heterodimer that binds and ‘‘caps” the barbed end of actin filaments in a reversible and calcium-independent manner to limit growth of the actin filament at this end. It is a key regulator of action polymerization in a wide variety of tissues and organisms [21–23]. Therefore, a down-regulation of CapZ in vivo would allow the actin filaments to keep growing and result in the accumulation of actin filaments, which could alter regulation of a number of actin-binding activities as well as interfering with directed cell movement, cell polarity and signaling events. It would cause the cells to break apart and undergo lysis ultimately [24,25]. In comparison to H1N1pdm09-infected A549 cells, the expression of CAPZA1 (CapZ-a1), one of a subunit isoforms of CapZ [23], was down-regulated in H7N9-infected A549 cells at 24, 48 and 72hpi, which indicated that the H7N9 virus could seriously affect the synthesis of CAPZA1 in A549 cells.

Ornithine-δ-aminotransferase (OAT) is a nuclear encoded, pyridoxal-5'-phosphate (PLP)-dependent enzyme, found in the mitochondrial matrix of most human tissues [26,27]. It involves in the synthesis of glutamate from ornithine, and further synthesizes proline and glutamine, as well as regulating the levels of ornithine in the cells [28–30]. The formation of these products from ornithine is of great physiological importance for diverse functions, such as growth, development, and response to a wide range of signaling molecules [31]. Wang et al [32] found that OAT has played a crucial role in regulating mitotic cell division. The decrease of OAT could block cell division and lead to cell death. Compared with the H1N1pdm09 infection, the down-regulation of OAT in H7N9-infected A549 cells at 24, 48 and 72hpi maybe block cell division and cause cell death.

Poly(rC)-binding protein 1 (PCBP1) is a member of the hnRNP family, which contains three K-Homology (KH) domains and two nuclear localization signals [33,34]. It is a RNA-binding and DNA-binding protein, as well as regulating transcriptional and translational processes [35]. It also involves in messenger RNA (mRNA) shuttling between the cytoplasm and nucleus, mRNA stability, protein-protein interactions and so on [36]. Huo et al [37] found that five pathways were recognized as being significantly impacted by the overexpression of exogenous PCBP1, including the translation factors pathway, hypertrophy model pathway, cell cycle pathway, integrin-mediated cell adhesion pathway and apoptosis mechanism pathway. Furthermore, they found that PCBP1 was a positive regulator for the cell cycle and anti-apoptosis. We observed that the levels of PCBP1 in H7N9-infected A549 cells were down-regulated at 24h and 48h as compared to the H1N1pdm09 infection, which inhibited cell cycle and induced apoptosis.

Eukaryotic translation initiation factor 5A (EIF5A), a nucleocytoplasmic shuttle protein, is the only protein containing hypusine [Ne-(4-amino-2-hydroxybutyl)lysine], which is essential for EIF5A function [38]. The protein is relate to eukaryotic translation [39], cell viability [40] and cell proliferation [41]. Maier et al [42] identified EIF5A as a critical regulator of the inflammatory response. Notably, EIF5A is a major factor that controls the balance between cell proliferation and death. Moreover, the depletion of EIF5A would exert strong antiproliferative effects in mammalian cells, leading to the inhibition of cell proliferation by arresting the cell cycle at the G1/S boundary [43,44]. In the present study, we found that EIF5A was prominently down-regulated at 48h and 72h in H7N9-infected A549 cells.

Platelet-activating factor acetylhydrolaze (PAFAH) can inactivate the potent pro-inflammatory phospholipid platelet-activating factor (PAF) by removing the acetyl group at its sn-2 position. This family of enzymes is comprised of one secreted (plasma) and at least four intracellular isozymes (isoforms Ⅰa, Ⅰb, Ⅱ, erythrocyte form) [45,46]. Isoform Ⅰb is a G-protein-like complex with two catalytic subunits (α1 and α2) and a regulatory β subunit, which inactivates PAF during its formation, and thus controls the secretion of active PAF and also acts by inhibiting intracellular functions of PAF [47–49]. The PAF mediates an array of biological processes (fertilization, fetal development, blood pressure). It also appears to be involved in pathological processes (inflammation, allergies, neural disorders, asthma, and chronic obstructive pulmonary disease) that affects the cardiovascular, cerebral, respiratory, gastrointestinal systems. It is worth noting that the PAF can stimulate the host to produce inflammatory cytokines, induce platelet aggregation and vascular permeability [45,50].

Our study showed platelet-activating factor acetylhydrolaseⅠb subunit beta (PAFAH1B2)to be significantly down-regulated at 72h in H7N9-infected A549 cells. The level of PAF would be up-regulated, which was related to the later clinical symptoms of patients infected with H7N9 virus, such as pneumonia, ARDS, encephalopathy and so on. Moreover, the increase of PAF induces large amounts of platelets to aggregation which can produce a wide range of micro thrombus in the host. It maybe the reason that the patients infected with H7N9 virus exhibit thrombocytopenia and coagulopathy [5]. In addition, the up-regulation of PAF is also consistent with previous findings showing that acute-phase serum samples from H7N9-infected patients contain elevated levels of inflammatory cytokines and induce vascular permeability [6,9,11].

We utilized proteomic approaches to identify differential expressions of cellular proteins in A549 cells infected with H7N9 and H1N1pdm09 influenza virus at 24, 48, 72hpi. It suggests that these differentially expressed proteins are involved in the pathogenic mechanisms of H7N9 infection at the cellular level. A study by Simon et al [15] infected A549 human cells with seasonal H1N1 (sH1N1), H1N1pdm09, or H7N9 and HPAI H5N1 strains(MOI of 10) to measure proteomic host responses to these different strains at the early stage of 1, 3, and 6hpi. However, their study showed that PCBP1 and EIF5A were up-regulated in A549 cells infected by the H7N9 virus and were not affected in the cells infected by H1N1pdm09 virus at 6hpi. These may be the cells’ stress response at the early stage of H7N9 infection in order to keep cell viability and anti-apoptosis. Morrison et al[9] considered that the depression of coagulation factor transcription in the lungs of mice infected with H7N9 virus was the reason of infection-induced vascular permeability and coagulopathy. While the decreasing of PAFAH1B2 in H7N9 infection maybe another reason of infection-induced vascular permeability and coagulopathy in our study. We first find that the expression of PAFAH1B2 is down-regulated significantly in H7N9 infection, which may provide a new therapeutic direction for the patients infected by H7N9 influenza virus. We will carry out further research to elucidate the mechanisms of PAFAH1B2 in H7N9 infection.
